# A multi-institutional approach to data-driven decision-making: National Evaluation Platform in Mali

**DOI:** 10.7189/jogh.09.010315

**Published:** 2019-06

**Authors:** Youssouf Keita, Niakalé Diawara, Bakary Diarra, Hamadoun Sangho, Harouna Koné, Mamadou Souncalo Traoré, Mama Coumaré, Ousmane Ly, Assa Sidibé Keita, Haoua Dembélé Keita, Ibrahim Téréra, Mariam Traoré, Talata Sawadogo-Lewis, Emilia Vignola, Rebecca Heidkamp, Melinda Munos, for the Mali NEP Working Group

**Affiliations:** 1 Institute for International Programs, Johns Hopkins University Bloomberg School of Public Health, Bamako, Mali; 2 Institut National de Recherche en Santé Publique (INRSP), Bamako, Mali; 3 Centre de Recherche, d’Etudes et de Documentation pour la Survie de l’Enfant (CREDOS), Bamako, Mali; 4 Institut National de la Statistique (INSTAT), Bamako, Mali; 5 Ministère de la Santé et de l’Hygiène Publique (MSHP), Bamako, Mali; 6 Agence Nationale de Télésanté et d’Informatique Médicale (ANTIM), Bamako, Mali; 7 Institute for International Programs, Johns Hopkins University Bloomberg School of Public Health, Baltimore, Maryland, USA

The use of evidence for decision-making around maternal, newborn, and child health and nutrition (MNCH&N) policies and programs calls for data and skills belonging to different institutions and sectors and requires effective collaboration between data, policy and program stakeholders in countries. Country governments and their partners must make many decisions about how to use scarce health resources. Although many countries have data that could be brought to bear on these decisions, such as household and health facility surveys, routine health information systems, and various program monitoring and evaluation efforts, these data are not always able to be used effectively for decision-making. Data may be owned by different groups within and outside Ministries of Health; data owners do not always have the capacity to analyze their data or interpret the results; and data may not be collected at or disaggregated to levels that are useful for decision-making.

The National Evaluation Platform (NEP) aims to build the capacities of and tools for national institutions to use data from various sources for better program evaluation and decision-making [1]. NEPs support government efforts to plan and evaluate the implementation and impact of large-scale MNCH&N programs, plans, and strategies. The NEP brings together institutions engaged MNCH&N data, policy-making, and program implementation to do collaborative work – posing policy-relevant evaluation questions, assembling and analyzing data and producing outputs for decision-maker audiences.

Mali is one of four sub-Saharan African countries that established an NEP in 2014 with funding from Global Affairs Canada and technical support from the Institute for International Programs of Johns Hopkins University (IIP-JHU) [2]. In an effort to address the siloing of MNCH&N data and expertise in Mali, and in response to feedback from the Ministry of Health and Public Hygiene (MoHPH), NEP-Mali established a multi-institutional, collaborative structure based around five governmental institutions. At the outset, we anticipated that the large number of institutions engaged in NEP in Mali would present logistical and technical challenges. However, we found that the NEP-Mali structures and funding were effective in facilitating collaboration between these institutions. We describe the establishment and functioning of the NEP-Mali structures and the advantages and challenges of this approach for evidence-based decision-making in Mali – and potentially in other settings.

## OBJECTIVES, STRUCTURE AND FUNCTIONING OF THE NEP-MALI

**Figure 1:**
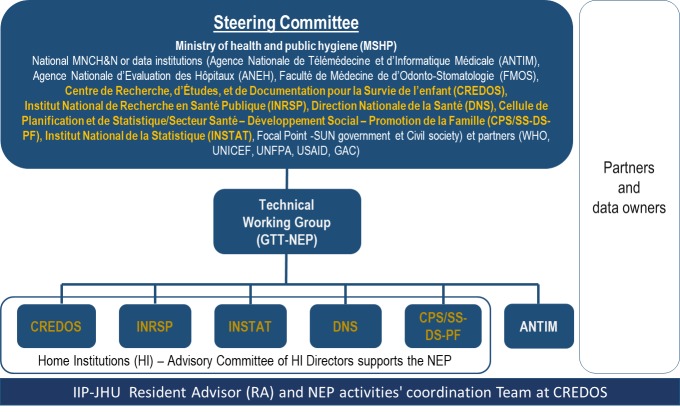
National Evaluation Platform (NEP) – Mali structure.

The NEPs in all four countries had the same basic organizational structure that aimed to bring together institutions that have not historically collaborated. They were comprised of a Steering Committee (SC) engaging higher-level MNCH&N decision-makers, a Technical working group (TWG) composed of individuals with MNCH&N program and/or data expertise, and a Home Institution (HI) responsible for coordination and oversight of NEP functions [1]. **Figure 1** illustrates the structure of NEP-Mali.

### Selection of home institutions

Mali formally designated five institutions as HIs: the National Institute of Research in Public Health (INRSP), the Centre for Research, Studies and Documentation for Child Survival (CREDOS), the Planning and Statistics Unit/ Health Sector – Social Development and Family Promotion (CPS/SS-DS-PF), the National Directorate of Health (DNS), and the National Institute of Statistics (INSTAT) which is part of the ministry of land and population planning.

The selection process for HIs for included meetings between the IIP-JHU team, Global Affairs Canada (GAC), and in-country research, planning, and implementing institutions; use of a questionnaire to assess technical and managerial capacity; and discussions between stakeholders [3]. Initially, stakeholders suggested three different institutions as HIs for NEP-Mali, namely CREDOS, INRSP, and INSTAT – CREDOS for their expertise in newborn and child health; INRSP for their broad public health and nutrition research expertise; and INSTAT for their statistical capacity. Because each institution had complementary and necessary areas of expertise, NEP-Mali was established with three HIs instead of only one. NEP-Mali’s SC then recommended that the DNS and the CPS/SS-DS-PF be added as home institutions. DNS is an implementing institution and CPS/SS-DS-PF is a stakeholder in program planning. Both are involved in the management of health data collected by MoHPH.

CREDOS, INRSP, and INSTAT were each funded separately by IIP-JHU, with CREDOS managing the funds for meetings and workshops and funding participants from the DNS and CPS/SS-DS-PF.

### Steering committee

A ministerial decision promulgated by the MoHPH formalized the Steering Committee (SC) [4]. The SC’s mission was to validate proposed MNCH&N research and evaluation questions for the NEP, facilitate the Technical Working Group’s (TWG) access to data and information, receive the results of the TWG’s analyses, validate the results, and ensure their wide dissemination and use at various decision-making levels. The SC brought key MNCH&N stakeholders together, including national institutions working in research, program implementation, and evaluation; academia; UN institutions; and civil society. The SC met on average every three months under the chairpersonship of the Secretary General of the MoHPH.

### Technical Working Group

The TWG was the engine of the NEP-Mali, tasked with examining proposed evaluation questions, carrying out analyses for validated questions, and presenting results. TWG members were drawn from the five home institutions, with 15% to 100% of their level of effort dedicated to and corresponding salary support provided by the NEP. To form the TWG, we specified a range of desired profiles (eg, expertise in MNCH&N, in analysis of household survey data, or in routine health systems data) and requested that HIs propose corresponding participants. This process resulted in varied and complementary profiles within the TWG. Each TWG member was designated their home institution to ensure his or her stability in the group and participation in TWG activities. Initially each HI had two members on the TWG, which later increased to 3 in anticipation of turnover. Since 2014, only two members of the TWG have left, one due to retirement and one for personal reasons.

### Additional structures

A coordination team composed of 6 individuals from CREDOS including the PI, a focal point, a national coordinator, a data manager, and the JHU Resident Advisor monitored NEP activities, issued weekly progress assessments, and coordinated report production.

To facilitate implementation, the five HIs directors established an advisory committee that were scheduled to meet monthly. These meetings addressed data requests, TWG member availability, and institutionalization of the NEP. The group also reviewed and refined evaluation questions and reviewed the results achieved by the TWG prior to submission to the SC.

## OPERATION OF THE NEP-MALI

### Question development and validation

Priority questions to be addressed by NEP-Mali could be proposed by the SC, the TWG, or other MNCH&N partners in Mali. There were up to three steps for the validation of proposed questions:

**Step 1:** TWG members examined the question, considering the data and analytical methods required. If the TWG judged that the question could be addressed, they prepared a summary of the data, methods, tools and skills needed to address it, and submitted it to the Home Institution directors.

**Step 2:** The directors of the HIs, upon receiving a question from the TWG, judged the relevance for the country and decided to adopt, adjust, or abandon it.

**Step 3:** When a question passed through the two previous steps, it was submitted to the SC, which decided whether to validate the question.

### Addressing priority questions

After the validation of priority questions by the SC, the TWG took ownership of the questions. For data that were not in the public domain, the TWG issued formal data requests to the relevant institutions. Where necessary, the TWG then undertook data quality assessments. The TWG and coordination team also worked with IIP-JHU to identify and develop a strategy to address capacity-building needs.

**Figure:**
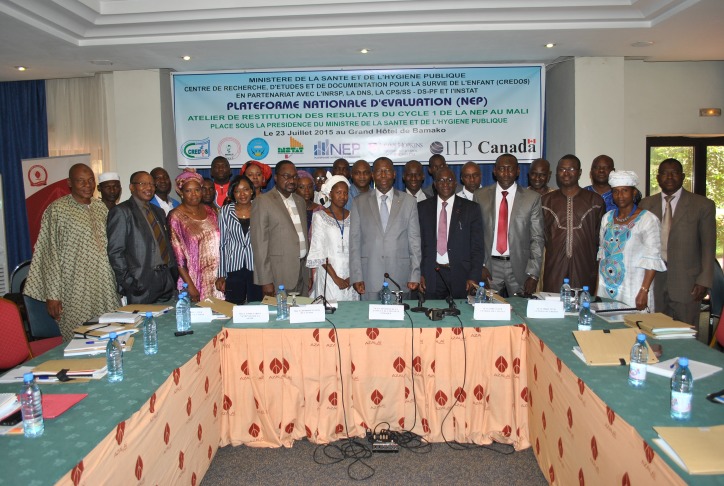
Photo: NEP-Mali Steering Committee and select Technical Task Team members meet for the dissemination of Cycle 1 results in Bamako, Mali (from the Team’s own collection, used with permission).

Capacity-building strategies included short (3-5 days) capacity-building workshops, ongoing mentorship of a TWG sub-group carrying out the analysis, and/or statistical/data management support by members of the IIP-JHU team. Capacity-building was enhanced by the availability of a team of data analysts and statisticians at IIP-JHU as well as an online tool for sharing and modifying analytical code (Stats Report; statsreport.io [5]). We conducted approximately 13 capacity-building workshops from 2013 to 2017. Between workshops the TWG met twice per month to advance work. Sub-groups continued this work between meetings.

## IMPACT OF THE NEP IN MALI

As the final reports of the NEP-Mali are still being developed, the full impact cannot be assessed at this time; however, we note several important results:

**On MNCH&N programs:** External evaluation firm FSG concluded in their 2017 evaluation of the NEP [6] that two NEP results had direct impacts on policy: (1) NEP Cycle 1 findings highlighted discrepancies in the coverage targets used across various MNCH&N-related plans, leading to a HLAC discussion of the need to create more coherent and realistic plans for MNCH [6] and (2) the SC and the MoHPH recommended that DNS and CPS/SS-DS-PF utilize the results of the NEP-Mali Lives Saved tool (LiST) analysis to improve MNCH&N plans [7-10].

**On the health system:** The SC and the MoHPH recommended that the NEP team train regional and district health staff on capacity building & use of LiST for planning to improve planning of MNCH&N programs [5]. A proposal has been developed by the NEP-Mali team and has received funding from the government of Canada, and training is scheduled to take place in 2018. LiST was incorporated in the University of Bamako’s public health master program, and the National Unit for the Nutrition Multi-Sectoral Coordination Chief of Party requested that NEP lead the establishment and management of the research and academic network of the Scaling Up Nutrition (SUN) movement in Mali [10].

**On the NEP approach itself**: The MoHPH asked the NEP HIs to propose a technical note for the institutionalization of the NEP in Mali. As part of the process of institutionalization, in 2018, CREDOS included budget line to support NEP-Mali’s work.

## ADVANTAGES AND CHALLENGES OF THE NEP-MALI APPROACH

### Advantages

The multi-institutional nature of the NEP had several positive impacts on the NEP in Mali. One of these was improved access to data, including survey and routine health systems data, as well as official documents and plans. It also resulted in a TWG whose members represented multiple complementary areas of expertise: epidemiologists, statisticians, pediatricians, nutritionists, and experts in Mali’s routine health information system. This membership allowed the TWG to effectively address the NEP’s evaluation questions. In addition, official designation of TWG members, provision of salary support, focus on capacity-building, and bi-monthly TWG meetings encouraged consistent participation and engagement by TWG members in the NEP-Mali’s work. The engagement of the directors of the HIs, who have acted as champions of the NEP in Mali, was also key to facilitating the work of the TWG and driving interest in institutionalizing the NEP in Mali.

Despite inconsistent participation of SC members, the presence of key MNCH&N institutions in the SC facilitated ownership of NEP results by decision-makers. In addition, the chairpersonship of the SC by the MoHPH strongly facilitated stakeholders’ buy-in to the NEP’s results.

### Challenges

Despite anticipating that coordinating five HIs would present a challenge, once the NEP structures were in place we encountered no coordination problems. The primary challenges of the NEP-Mali related to SC member turnover, as well as external support for the NEP. Because SC membership was institutional rather than nominative and many institutions experienced substantial turnover since 2013, there were substantial changes in SC composition from meeting to meeting. The HI directors and the academic representative were an important source of institutional knowledge for the SC.

Financial sustainability of the NEP post-GAC funding is an ongoing concern. Although a HI was successful in creating budget line that cover a portion of the cost of the NEP, we have been unable to identify a complementary source of external funding to allow NEP activities to continue.

## CONCLUSION

Involving five different institutions was initially logistically complex, but ultimately facilitated NEP-Mali’s work and increased its influence. The presence of several strong research institutions in Mali with a good level of research capacity made this approach possible; we note that this may not be the case in all settings. Given the success of the multi-institutional approach for NEP-Mali, this model could be applied to other sectors in Mali as well as in other countries with a similar profile.
